# Conjunctival transforming growth factor-β2 and vascular endothelial growth factor in canine keratoconjunctivitis sicca: Baseline alterations, clinical associations, and response to 0.2% cyclosporine therapy

**DOI:** 10.14202/vetworld.2026.295-309

**Published:** 2026-01-25

**Authors:** Bianca Eidt Rodrigues, Alexandre Pinto Ribeiro, Tiago Barbalho Lima, Alcyjara Rego Costa, Marvin Paulo Lins, Luis Jhordy Alfaro Quillas

**Affiliations:** 1 Faculdade de Medicina Veterinária, Universidade Federal de Mato Grosso, Mato Grosso, Cuiabá, Brazil; 2 Departamento de Clínica Veterinária, Hospital Veterinário Universitário, Universidade Estadual do Maranhão, São Luíz, Maranhão, Brazil; 3 Departamento de Ciências Básicas em Saúde, Faculdade de Medicina, Universidade Federal de Mato Grosso, Cuiabá, Brazil

**Keywords:** canine keratoconjunctivitis sicca, cyclosporine therapy, goblet cell density, ocular surface inflammation, tear film biomarkers, transforming growth factor-β2, vascular endothelial growth factor, veterinary ophthalmology

## Abstract

**Background and Aim:**

Transforming growth factor-β2 (TGF-β2) and vascular endothelial growth factor (VEGF) are key mediators of inflammation, fibrosis, and angiogenesis in ocular surface disease. However, their roles in canine keratoconjunctivitis sicca (KCS) are not well understood. This study aimed to compare conjunctival TGF-β2 and VEGF levels between healthy dogs and those with KCS, evaluate the effects of 6-week therapy with 0.2% cyclosporine A (CsA), and explore associations with clinical signs, Schirmer tear test-1 (STT-1), goblet cell density (GCD), and inflammatory cell infiltration.

**Materials and Methods:**

Thirty-three dogs with KCS, classified as mild (n = 10), moderate (n = 10), or severe (n = 13), underwent ophthalmic exams, STT-1 measurements, and conjunctival biopsies before treatment (T0) and after 6 weeks of topical CsA therapy (T1). Fourteen healthy dogs served as controls. Conjunctival samples were analyzed for GCD, inflammatory cell counts, and TGF-β2 and VEGF levels using histology and enzyme-Linked Immunosorbent Assay. Clinical scoring and corneal vascular quantification were performed using standardized protocols. Statistical comparisons were made within and between groups, as well as through correlation analyses.

**Results:**

CsA significantly increased STT-1 in all KCS grades and improved selected clinical signs. GCD in KCS dogs increased at T1, reaching levels comparable to controls, although not statistically significant. Neutrophils were the only inflammatory cells to significantly decrease after treatment. Overall, TGF-β2 levels did not differ between controls and KCS dogs; however, concentrations increased with disease severity and showed a positive correlation with lymphocyte counts and corneal melanosis, and a negative correlation with GCD. VEGF levels were mildly elevated in KCS but decreased significantly following CsA treatment, especially in severe cases, and correlated positively with corneal melanosis and negatively with corneal vessel counts. A positive correlation was observed between TGF-β2 and VEGF.

**Conclusion:**

Topical 0.2% CsA improves tear production, GCD restoration, and various clinical signs in canine KCS. TGF-β2 seems to have a pro-inflammatory and profibrotic role, increasing with disease severity and linked to chronic ocular surface changes. CsA effectively decreases VEGF, especially in severe KCS, indicating partial modulation of angiogenic pathways. Longer treatment durations may be necessary to influence TGF-β2-mediated tissue remodeling.

## INTRODUCTION

The pathophysiology of canine keratoconjunctivitis sicca (KCS) closely resembles that of dry eye disease (DED) in humans with Sjögren’s syndrome (SS) [1]. Both conditions involve chronic inflammation of the lacrimal glands, leading to significant reductions in aqueous tear production, damage to the ocular surface, and discomfort. In SS, autoimmune exocrinopathy, T-lymphocyte infiltration, and destruction of the lacrimal and salivary glands lead to xerophthalmia and xerostomia [2–6]. Similarly, in canine immune-mediated KCS, lymphocytic-plasmacytic infiltration of the lacrimal and nictitans glands is believed to result from a type IV hypersensitivity reaction, leading to glandular atrophy and tear film deficiency [1]. CD4+ T cells are mainly responsible for driving glandular destruction and maintaining inflammation in both species [2–6]. Therefore, the strong immunopathological similarities between SS in humans and immune-mediated KCS in dogs support the use of canine KCS as a valuable model for studying autoimmune DED in humans [1].

The conjunctival goblet cells are responsible for producing and secreting mucin, which aids in attaching the tear film’s aqueous layer to the corneal surface [7,8]. Although goblet cell density (GCD) assessment is not necessary for diagnosing quantitative KCS, two studies based on conjunctival biopsy have reported GCD changes associated with disease progression [7,8]. In most cases, KCS is effectively managed with topical cyclosporine A (CsA) or tacrolimus, both of which improve clinical signs and increase Schirmer tear test-1 (STT-1) and GCD values [2–6].

Transforming growth factor-beta (TGF-β) is a versatile cytokine that can have either pro-inflammatory or anti-inflammatory effects depending on the context. It inhibits extracellular matrix breakdown by suppressing metalloproteinases and serine proteases while increasing protease inhibitors, which ultimately leads to fibrosis [9]. TGF-β1 and TGF-β2 have different distribution patterns in the human anterior segment [9]. The lacrimal gland produces TGF-β1 [10], whereas conjunctival goblet cells release TGF-β2 [11]. The role of TGF-β in DED remains debated [11, 12]. In one experimental study, blocking TGF-β signaling in CD4+ T cells improved DED symptoms in mice subjected to desiccating stress [12]. Conversely, in humans with DED, conjunctival TGF-β2 levels are significantly lower than in healthy controls and increase after two months of topical cyclosporine A (CsA) treatment [11]. No research has examined conjunctival TGF-β2 in dogs with keratoconjunctivitis sicca (KCS), and the effects of CsA on this cytokine in dogs are still unknown.

Vascular endothelial growth factor (VEGF) is a powerful angiogenic and endothelial-specific mitogen. The VEGF family includes VEGF-A, -B, -C, -D, the viral homolog VEGF-E, and placental growth factor. VEGF is a key mediator of angiogenesis, with increased expression during neovascularization and a crucial role in inflammatory, neoplastic, and vascular ocular diseases [13, 14]. In dogs with KCS, corneal neovascularization decreases visual acuity and promotes melanocyte migration, which can lead to blindness [1–6]. In experimental mouse models, desiccating stress raises VEGF-A levels and stimulates lymphangiogenesis through VEGF-C and VEGF-D [15, 16]. However, no significant changes in tear VEGF levels have been observed in patients with allergic disease or DED-SS [15]. Higher VEGF levels in platelet-rich plasma eye drops used in DED-SS are associated with better symptoms and increased STT-1, indicating a potential role in ocular surface healing [17].

Topical CsA, a calmodulin inhibitor that suppresses CD4+ T-cell activity, improves tear production, increases conjunctival GCD, and alleviates clinical signs in most dogs with KCS [2–5]. One study reported that 0.1% CsA was more effective than bevacizumab in reducing corneal edema, vascularization, and inflammation while promoting more organized collagen in alkali-burned rat corneas [18]. The expression of VEGF and its receptors (Flt-1 and Flk-1) has been observed in human corneas affected by infection, atopy, trauma, and chemical burns [13]. A review identified 38 molecules potentially involved in the inflammatory response of DED [19]. However, no studies have evaluated conjunctival VEGF in humans or dogs with DED or KCS.

Previous investigations into the management of canine keratoconjunctivitis sicca (KCS) have primarily focused on clinical responses to immunomodulatory therapy, especially tacrolimus and cyclosporine A (CsA). Early studies demonstrated that topical tacrolimus increases tear production and improves clinical signs in affected dogs [2, 3]. Later comparative studies confirmed that both tacrolimus and CsA are effective in raising Schirmer tear test values and reducing ocular inflammation [3–5]. However, these studies only assessed clinical or gross histological outcomes, leaving the underlying molecular mechanisms unexplored.

Research on conjunctival changes during CsA therapy has been mainly limited to morphological studies. For instance, improvements in GCD and inflammatory cell populations induced by CsA were documented using conjunctival biopsy, yet no analysis of cytokine expression or signaling pathways was conducted [4]. Likewise, studies comparing CsA and tacrolimus did not examine biochemical mediators that might contribute to chronic inflammation, fibrosis, or angiogenesis in KCS [3–5]. Investigations using impression cytology have offered insights into conjunctival metaplasia and epithelial recovery during treatment [20], but these results primarily reflect structural alterations rather than the underlying molecular mechanisms.

No published research has investigated whether KCS is linked to changes in conjunctival levels of transforming growth factor-β2 (TGF-β2) or VEGF, despite the critical roles these cytokines play in controlling inflammation, tissue remodeling, goblet cell differentiation, and new blood vessel formation. Additionally, the possible effect of topical CsA on these biomarkers in dogs has not been explored. Consequently, the cytokine-driven mechanisms that influence disease severity, chronicity, and treatment response in KCS are still not well understood.

This lack of cytokine-level research represents a significant gap in understanding the biological basis of ocular surface changes in KCS and limits our ability to explain why some clinical signs resolve quickly while others, like melanosis or vascularization, worsen despite treatment.

The present study aimed to clarify the molecular and clinical mechanisms behind canine keratoconjunctivitis sicca (KCS) by examining cytokine expression patterns in the conjunctiva. Specifically, this research sought to determine whether transforming growth factor-β2 (TGF-β2) and VEGF, two important regulators of inflammation, fibrosis, goblet cell function, and angiogenesis—are altered in dogs with KCS compared to healthy dogs. Another goal was to assess whether a 6-week treatment with topical 0.2% cyclosporine A (CsA) affects conjunctival cytokine levels, providing insight into the biochemical response to standard immunomodulatory therapy. Additionally, the study aimed to explore how TGF-β2 and VEGF relate to clinical severity, Schirmer tear test-1 (STT-1) results, GCD, and inflammatory cell infiltration, with the goal of identifying cytokine signatures linked to ocular surface dysfunction. By combining clinical scoring, histological evaluation, and quantitative cytokine analysis, this research strives to develop a more comprehensive understanding of the inflammatory and angiogenic pathways involved in KCS and to address the scarcity of molecular data on the ocular surface response to CsA treatment.

## MATERIALS AND METHODS

### Ethical approval

This study was conducted in accordance with the Animal Research: Reporting of *In Vivo* Experiments (ARRIVE) 2.0 guidelines and the Guide for the Care and Use of Laboratory Animals (NIH, 2011). Ethical approval was received from the Ethics Committee of the Federal University of Mato Grosso (UFMT), Brazil (Protocol No. 23108.098827/2022-66), and the Ethics Committee of the State University of Maranhão (UEM), Brazil (Protocol No. 32/2023). All dogs involved in the study were privately owned, and written informed consent was obtained from the owners prior to inclusion. All clinical and ophthalmological examinations adhered to best veterinary practices. Painful procedures, such as conjunctival biopsies performed in an outpatient setting, were conducted under topical anesthesia, while biopsies from control dogs were performed under general anesthesia.

### Study design and treatments

This prospective study involved dogs admitted to the ophthalmology service at the Veterinary Teaching Hospitals of UFMT and UEM between January 2023 and December 2024. Inclusion criteria included a Schirmer tear test-1 (STT-1) result of ≤15 mm/min and at least one clinical sign, such as ocular discharge, conjunctival hyperemia, or corneal abnormalities like edema, vascularization, and melanosis. The severity of kerato-conjunctivitis sicca (KCS) was graded based on STT-1 readings into three categories: mild (11–14 mm/min), moderate (6–10 mm/min), and severe (0–5 mm/min) [1, 7]. Only dogs with the immune-mediated form of KCS and no prior ophthalmic treatment were included.

The exclusion criteria included the presence of concomitant ocular diseases such as glaucoma, uveitis, or corneal ulcers, previous ophthalmic surgery, and prior treatment with topical immunosuppressants. Other forms of KCS associated with infectious diseases (visceral leishmaniasis and distemper) were excluded based on polymerase chain reaction and serology results. Neurogenic KCS was ruled out through clinical evaluation of the facial and trigeminal nerves. Systemic disorders known to affect tear secretion, such as diabetes mellitus (urinary and serum glucose), hypothyroidism (serum triiodothyronine, thyroxine, and thyroid-stimulating hormone), and hyperadrenocorticism (dexamethasone suppression test), were also excluded.

Fourteen healthy dogs with no eye problems, no recent use (within the last 30 days) of systemic or topical eye medications, and STT-1 values over 15 mm/min served as controls.

At the first appointment (T0), selected patients with KCS received one drop of 0.2% cyclosporine A (CsA) every 12 h as the main treatment and one drop of 0.15% sodium hyaluronate six times daily as an adjuvant therapy until the first recheck, which occurred after a median period of 45 days (T1). Owners received detailed instructions regarding medication administration, and compliance was monitored through weekly telephone follow-ups.

The 0.2% CsA concentration in a sesame oil vehicle was chosen because it is available as a commercial veterinary formulation and previous studies have shown that concentrations between 0.05% and 2% are effective for treating canine KCS [2–5]. The six-week follow-up period was selected based on earlier research demonstrating notable increases in tear production and GCD at this time point [3–5], enabling the assessment of early treatment effects on cytokine modulation.

### Ophthalmic examinations

All patients received a clinical evaluation, including visual inspection, slit-lamp biomicroscopy, indirect ophthalmoscopy, tear volume measurement, intraocular pressure assessment (Tonovet®, iCare, Helsinki, Finland), and fluorescein staining. The clinician responsible for assessing the clinical signs at both T0 and T1 was blinded to STT-1 values and group assignment to prevent bias.

A modified scoring system was used to quantify macroscopic findings during slit-lamp biomicroscopy [21]. Corneal edema was scored as follows: 0 ≤ 25%, 1 = 26–50%, 2 = 51–75%, and 3 ≥ 76% of the corneal surface. Melanosis was expressed as a percentage of the affected area. Conjunctival hyperemia and ocular discharge were scored on a scale from 0 (none) to 3 (severe) [21].

For standardized documentation, the same evaluator captured images of the ocular surface at T0 and T1 using a smartphone (Samsung S24 Ultra®, Samsung, Noida, India) under consistent conditions, including a 12-megapixel resolution, built-in LED flash, no digital zoom (1× zoom), and automatic focus mode. The images were anonymized and evaluated blindly using a randomization platform (http://www.randomization.com).

**Figure 1:**
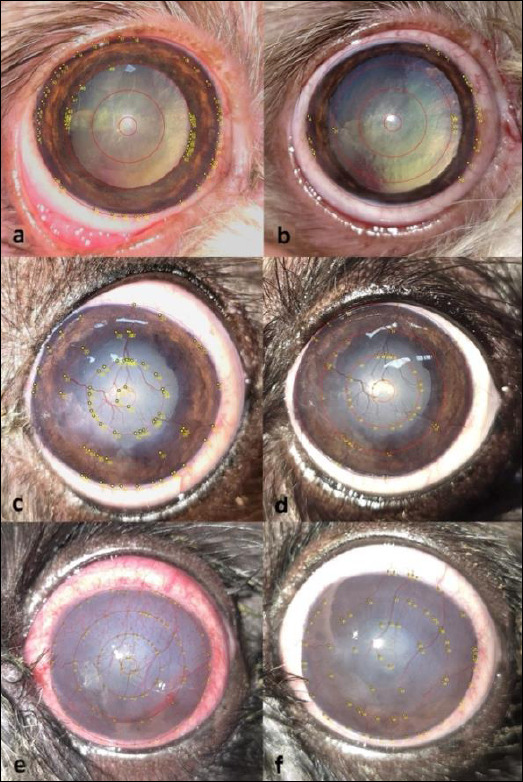
Representative images showing corneal vessel branches in dogs with (a and b) mild, (c and d) moderate, and (e and f) severe keratoconjunctivitis sicca (KCS) before treatment (a, c, and e) and after treatment (b, d, and f) with 0.2% cyclosporine. Differences among mild, moderate, and severe KCS groups for Schirmer tear test-1 (STT-1) values were analyzed using one-way analysis of variance followed by Bonferroni’s multiple comparisons test (a, b, and c differ significantly from each other). Differences among severity groups for clinical scores were assessed using the Kruskal–Wallis test followed by Dunn’s multiple comparisons test, with a and b differing for ocular discharge, a differing from the other two groups for corneal edema, and a, b, and c differing for corneal vessel branching. Differences between time points (T0 vs. T1) for STT-1 were analyzed using the paired t-test, while differences in ophthalmic clinical signs between time points were analyzed using the Wilcoxon test. Bold values indicate statistically significant differences (p < 0.05). KCS = keratoconjunctivitis sicca, STT-1 = Schirmer tear test-1, T0 = baseline, T1 = post-treatment.

Tools for quantifying corneal vascularization were manually applied as previously described in dogs [21]. Blood-filled vessel branches within predefined concentric circles were counted at T0 and T1 (Figure 1). The corneal surface was divided into four concentric circles using an ImageJ plugin, and the average number of vessel branches across these circles was calculated [21]. Analyses were conducted with ImageJ software (version 1.54p). Fluorescein staining was performed at both examinations. The median interval between T0 and T1 was 45 days.

To prevent bias, the examiner conducting the clinical assessments at both visits was blinded to STT-1 values.

### Conjunctival biopsy and determination of inflammatory cell count

In dogs with KCS, the ocular surface was rinsed with a 0.5% povidone–iodine solution at the end of each examination (T0). One drop of 0.5% proxymetacaine was instilled, and after 2 minutes, a conjunctival specimen (3.0–4.0 mm) was collected from the ventral-nasal fornix using toothed Catalano forceps and tenotomy scissors. The same procedure was repeated 45 days after treatment (T1), with sampling from an adjacent area identified by the visible scar from the first biopsy to minimize variability.

In control dogs, biopsies were taken once under general anesthesia. Dogs were pre-medicated with 0.3 mg/kg of methadone, anesthesia was induced with 5.0 mg/kg of intravenous propofol and maintained with inhaled isoflurane in 100% oxygen. One drop of 0.5% proxymetacaine was administered before biopsy collection. Postoperative pain relief included meloxicam (0.1 mg/kg) once daily for 2 days straight. Owners were contacted to monitor for bleeding or pain (such as blepharospasm or eye rubbing). Ophthalmic treatments started only after the postoperative period.

Half of each biopsy was used to quantify GCD and inflammatory cells, while the remaining half was stored in 1% protease-inhibitor solution at −80°C for later analysis of transforming growth factor-β2 (TGF-β2) and VEGF. The histological section was fixed in 10% buffered formalin, processed routinely, and stained with periodic acid–Schiff and hematoxylin–eosin. At ×40 magnification, the optimal field was selected, and nuclei from 50 basal epithelial cells were counted, along with their associated goblet cells. Lymphocytes, plasma cells, and neutrophils were counted in 10 high-power fields. All assessments were performed by a single examiner blinded to the treatment group and time point.

### Quantification of TGF-β2 and VEGF

Conjunctival samples were thawed at room temperature, and protein concentration was measured using the Bradford assay (Bio-Rad®, USA) to ensure suitability for ELISA. Homogenates were centrifuged at 10,000 × *g* for 15 minutes at 4°C, and supernatants were used for cytokine analysis. Commercial ELISA kits were employed to measure VEGF (FineTest®, China; Lot 46773025) and TGF-β2 (AFG Scientific®, USA; Lot 47004047) without dilution, according to the manufacturer’s instructions. Absorbance was read at 450 nm, and concentrations were calculated in pg/mL (VEGF) or ng/mL (TGF-β2) using four-parameter logistic curves (www.myassays.com). Detection limits and intra- and inter-assay coefficients of variation followed manufacturer specifications. Samples were anonymized and processed under blinded conditions.

### Statistical analysis

The sample size was calculated with a 5% alpha error and 80% power based on previously reported STT-1, inflammatory cell, and GCD data in dogs with KCS before and after CsA or tacrolimus treatment [3–6]. The Rosner test was used to identify outliers. Data normality was checked using the Shapiro–Wilk test. Parametric data were analyzed with unpaired and paired t-tests and analysis of variance (ANOVA) with post hoc tests. Non-parametric data were analyzed with the Wilcoxon test or Kruskal–Wallis test.

Differences in clinical signs between T0 and T1 were analyzed using the Wilcoxon test. STT-1, GCD, inflammatory cell counts, TGF-β2, and VEGF were analyzed using paired t-tests. Comparisons between control and KCS groups at T0 and T1 were conducted using unpaired t-tests. Differences among mild, moderate, and severe KCS were evaluated using the Kruskal–Wallis test (for clinical scores) or one-way ANOVA (for STT-1, TGF-β2, VEGF). Paired t-tests assessed cytokine changes within each severity category. Correlations were evaluated using Spearman or Pearson tests. The Fisher exact test assessed sex distribution between groups. Statistical significance was set at p < 0.05. Analyses were performed using R software (R Core Team, 2024).

## RESULTS

### Clinical signalment

A total of 33 dogs diagnosed with immune-mediated keratoconjunctivitis sicca (KCS) and treated with 0.2% cyclosporine A (CsA) were included in this study. Although KCS was diagnosed and treated in both eyes of some patients, only one eye per dog was used for statistical analysis. The study population included 17 males and 16 females, aged 2–15 years, who received CsA treatment for 40–61 days. Patients were classified into mild (n = 10), moderate (n = 10), and severe (n = 13) categories based on the degree of clinical severity.

The mild group included 7 males and 3 females, with an average age of 5.0 years (range 2.0–14.0), and they were treated for an average of 45.0 days (range 42.0–51.0). The breeds represented were Lhasa Apso (1), Shih Tzu (5), Schnauzer (1), and mixed-breed dogs (3). The moderate group consisted of 5 males and 5 females, with an average age of 9.5 years (range 3.0–15.0), treated for an average of 47.5 days (range 40.0–61.0). Breeds in this group included Lhasa Apso (1), Shih Tzu (7), Pug (1), and mixed-breed dogs (1). The severe group comprised 5 males and 8 females, with an average age of 4.0 years (range 2.0–14.0), and they were treated for an average of 45.0 days (range 40.0–61.0). Breeds included Lhasa Apso (1), Shih Tzu (8), and mixed-breed dogs (4).

Additionally, 14 dogs with at least one healthy contralateral eye were selected as controls. This population comprised 6 males (two castrated) and 8 females (two spayed), aged 2–14 years and weighing 3.8–25.4 kg. These dogs underwent surgical procedures including spay (n = 5), castration (n = 3), dental cleaning (n = 3), unilateral enucleation due to proptosis (n = 1), and correction of unilateral prolapse of the nictitating membrane gland (n = 2). The breeds represented were miniature Schnauzer (1), miniature Doberman Pinscher (3), Yorkshire Terrier (2), Shih Tzu (6), and Lhasa Apso (2). The Schirmer tear test-1 (STT-1) value of healthy control eyes was 24.14 ± 1.33 mm/min.

The number of males and females did not differ significantly among groups (*p* = 0.75). Ages did not differ significantly between controls (6.70 ± 1.12 years) and dogs with KCS (7.52 ± 0.50 years) (p = 0.43). Intraocular pressures were 13.50 ± 0.70 mm Hg in controls and 12.70 ± 0.51 mm Hg in dogs with KCS (p = 0.52).

### Ophthalmic signs and STT-1 expression

STT-1 values increased significantly from T0 to T1 (p < 0.05) across all stages of KCS. In the mild form, there were significant improvements in ocular discharge (p = 0.003) and conjunctival hyperemia (p = 0.003), while corneal vessel scores did not change significantly (p = 0.84) (Table 1). In the moderate and severe forms of KCS, no significant changes were observed in ocular discharge or conjunctival hyperemia (p > 0.05) (Table 1).

**Table 1:** Comparison of tear film parameters and ocular surface findings between healthy dogs and dogs with keratoconjunctivitis sicca (mean ± SEM).

Parameters	Mild (n = 10)	Moderate (n = 10)	Severe (n = 13)	p-values
STT-1				
T0	13.10 ± 1.19^a^	9.10 ± 0.87^b^	2.61 ± 2.53^c^	**<0.001**
T1	18.00 ± 3.59^a^	17.30 ± 3.81^b^	6.76 ± 4.88^c^	**<0.001**
p-value	**0.001**	**<0.0001**	**0.009**	
Discharge				
T0	2.00 (1.0–2.0)^a^	1.50 (0.0–3.0)	3.00 (1.0–3.0)^b^	**0.02**
T1	1.00 (0.0–1.0)^a^	1.00 (0.0–2.0)	2.00 (0.0–3.0)^b^	**0.03**
p-value	**0.003**	0.12	0.12	
Conjunctival hyperemia				
T0	2.00 (1.0–2.0)	1.50 (0.0–3.0)	3.00 (1.0–3.0)	0.45
T1	1.00 (0.0–1.0)	1.00 (0.0–2.0)	2.00 (0.0–3.0)	0.38
p-value	**0.003**	0.12	0.12	
Corneal edema				
T0	1.00 (0.0–2.00)	1.00 (0.0–3.00)	3.00 (1.0 – 3.00)^a^	**0.01**
T1	0.50 (0.0–1.00)	0.00 (0.0–3.00)	2.00 (1.0 – 3.00)^a^	**0.04**
p-value	**0.003**	**0.04**	0.16	
Corneal vessel branches				
T0	3.00 (0.0–24.0)^a^	11.0 (0.0–21.0)^b^	27.0 (3.0–100.0)^c^	**<0.001**
T1	2.00 (0.0–36.0)^a^	6.5 (0.0–25.0)^b^	19.50 (0.0–69.0)^c^	**<0.001**
p-value	0.84	0.09	**0.005**	
Corneal melanosis				
T0	1.50 (0.0–21.44)^a^	19.37 (0.0–99.18)^b^	23.02 (0.0 – 91.75)^c^	**<0.001**
T1	0.50 (0.0–25.36)^a^	17.77 (0.0 – 100.0)^b^	27.58 (0.0 – 93.40)^c^	**<0.001**
p-value	0.43	0.13	0.84	

Data are presented as numbers (%) or means ± SEM, as appropriate. Bold values indicate statistically significant results. Differences among mild, moderate, and severe KCS for STT-1 were assessed using a one-way analysis of variance followed by Bonferroni's multiple comparisons test (a, b, and c: Differ from each other). Differences among mild, moderate, and severe KCS for clinical scores were assessed using the Kruskal–Wallis test followed by Dunn's multiple comparisons test (a and b: Differ among each other for ocular discharge; a: Differ from the other two groups for corneal edema; a,b, and c: Differ among each other for the corneal vessel branches Differences between time points (T0 vs. T1) for STT-1 were analyzed using the paired t-test. Differences in ophthalmic clinical signs between time points (T0 vs. T1) were analyzed using the Wilcoxon test. KCS = keratoconjunctivitis sicca, STT = Schirmer tear test (mm/min), SEM = standard error of the mean.

A notable decrease in corneal vessel branches was seen only in the severe form (p = 0.005) (Table 1, Figure 1). No significant improvements in corneal melanosis were observed in any severity category (p > 0.05) (Table 1). The fluorescein test was negative for all patients at both time points.

### Conjunctival GCD and inflammatory cell count

Out of the 33 dogs with KCS, only 14 had conjunctival samples available for GCD measurement at both time points. Biopsies from dogs treated with 0.2% CsA were taken from mild (n = 4), moderate (n = 5), and severe (n = 5) cases.

**Figure 2:**
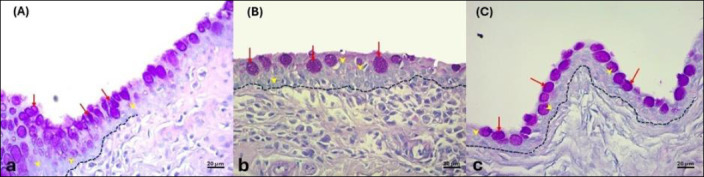
Representative photomicrographs showing conjunctival goblet cell density in (a) control eyes and (b) in eyes with keratoconjunctivitis sicca (KCS) before and (c) after treatment with 0.2% cyclosporine. The outlined dashed area represents a standardized field of 50 conjunctival epithelial cells (yellow arrowheads), within which goblet cells (red arrows) were counted. Sections were stained with acidic periodic Schiff and hematoxylin and eosin and examined at ×40 magnification. Comparisons between control and KCS-affected eyes were performed at both baseline (T0) and post-treatment (T1) using an unpaired t-test. Comparisons between time points (T0 vs. T1) within KCS-affected eyes were analyzed using a paired t-test. Bold values indicate statistically significant differences (p < 0.05). KCS = keratoconjunctivitis sicca, T0 = baseline, T1 = post-treatment.

At T0, the GCD of control eyes was significantly higher than that of dogs with KCS (p = 0.006) (Table 2, Figure 2). Although GCD increased at T1 after CsA treatment, the increase was not statistically significant compared to T0 (p = 0.46). However, at T1, the GCD of CsA-treated eyes was similar to that of control eyes (p = 0.20) (Table 2, Figure 2).

**Table 2:** Mean ± SEM of goblet cell density and numbers of lymphocytes, plasma cells, neutrophils, and total inflammatory cell counts in the conjunctiva of healthy control dogs and dogs with KCS before (T0) and after treatment (T1) with 0.2% cyclosporine.“

Parameter	Controls (n = 14)	KCS (n = 14)	p-values
Globlet cell density			
T0	96.14 ± 15.35	45.86 ± 7.56	**0.006**
T1	96.14 ± 15.35	68.43 ± 11.22	0.15
p-values (time points)	–	0.09	
Lymphocytes			
T0	2.96 ± 0.54	6.46 ± 0.94	**0.003**
T1	2.96 ± 0.54	8.56 ± 2.51	**0.04**
p-values	–	0.45	
Plasma cells			
T0	3.29 ± 0.46	18.43 ± 3.23	**0.0004**
T1	3.29 ± 0.46	15.56 ± 3.00	**0.001**
p-values	–	0.51	
Neutrophils			
T0	1.05 ± 0.23	2.83 ± 0.66	**0.02**
T1	1.05 ± 0.23	1.29 ± 0.23	0.47
p-values	–	**0.03**	
Total inflammatory cells			
T0	7.03 ± 0.80	36.43 ± 9.11	**0.0008**
T1	7.03 ± 0.80	25.67 ± 3.80	**0.0003**
p-values	–	0.22	

Values are presented as mean ± SEM. Different superscript letters within the same row indicate statistically significant differences (p < 0.05). Bold values are statistically significant (p < 0.05). Comparisons between groups (controls vs. KCS-affected eyes) were performed at both T0 and T1 using an unpaired t-test. Comparisons between time points (T0 vs. T1) within patients with KCS were analyzed using a paired t-test. KCS = keratoconjunctivitis sicca, TBUT = tear film breakup time, STT = Schirmer tear test, SEM = standard error of the mean.

The number of inflammatory cells in the conjunctiva of control dogs was significantly higher than in dogs with KCS at both time points (p < 0.05) (Table 2). Total inflammatory cells and plasma cells were lower at T1 compared to T0, but CsA did not significantly decrease these counts (p > 0.05). Lymphocyte counts increased from T0 to T1, but this change was not significant (p = 0.31). Neutrophils were the only inflammatory cells that significantly decreased after CsA treatment (p = 0.02) (Table 2).

### Conjunctival levels of TGF-β2 and VEGF

All 33 conjunctival samples from dogs with KCS were available for quantification of transforming growth factor-β2 (TGF-β2) and VEGF. In the control group, 11 of 14 samples were available for TGF-β2 measurement; one outlier (1,172.14 ng/mL) was excluded. For VEGF, 10 control samples were available.

**Figure 3:**
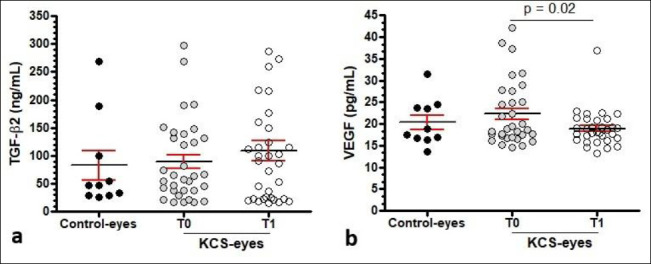
Conjunctival transforming growth factor-β2 and vascular endothelial growth factor levels in control and keratoconjunctivitis sicca–affected eyes before and after cyclosporine treatment. Scatter plots showing conjunctival concentrations of transforming growth factor-β2 (TGF-β2, ng/mL) and vascular endothelial growth factor (VEGF, pg/mL) in control eyes and in eyes with keratoconjunctivitis sicca (KCS) before treatment (T0) and after treatment (T1) with 0.2% cyclosporine. Individual data points are shown as circles, overall means are indicated by black horizontal lines, and error bars represent the standard error of the mean. Comparisons between control and KCS-affected eyes were performed at both T0 and T1 using an unpaired t-test. Comparisons between time points (T0 vs. T1) within KCS-affected eyes were analyzed using a paired t-test. Bold values indicate statistically significant differences (p < 0.05).

**Figure 4:**
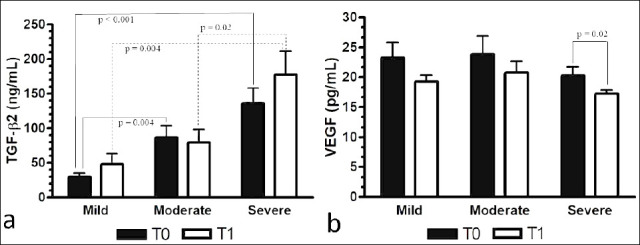
Severity-based changes in conjunctival cytokine levels before and after cyclosporine treatment in dogs with keratoconjunctivitis sicca. Mean ± SEM concentrations of transforming growth factor-β2 (TGF-β2, ng/mL) (a) and vascular endothelial growth factor (VEGF, pg/mL) (b) in eyes with mild (n = 10), moderate (n = 10), and severe (n = 13) keratoconjunctivitis sicca (KCS) before treatment (T0) and after treatment (T1) with 0.2% cyclosporine. At T0, cytokine levels in the moderate and severe KCS groups were significantly higher than those in the mild group. At T1, cytokine levels in the mild and moderate groups were significantly lower than those in the severe group, as indicated. Comparisons among KCS severity groups were performed using one-way analysis of variance with Bonferroni post hoc tests, and within-group changes between T0 and T1 were analyzed using paired t-tests. Bold values indicate statistically significant differences (p < 0.05). KCS = keratoconjunctivitis sicca, TGF-β2 = transforming growth factor-β2, VEGF = vascular endothelial growth factor, SEM = standard error of the mean, T0 = baseline, T1 = post-treatment.

TGF-β2 levels in the KCS group did not significantly differ from control eyes (p = 0.07) (Table 3, Figure 3a). Although TGF-β2 levels were higher at T1 after CsA treatment, the increase was not significant (p = 0.39). At T0, separate evaluations showed that TGF-β2 levels were significantly higher in moderate (p = 0.004) and severe cases (p < 0.001) compared to mild cases (Table 4, Figure 4a). Higher levels in severe cases versus moderate cases were observed but were not statistically significant (p = 0.10). At T1, TGF-β2 levels did not significantly differ from T0 within any severity category (p > 0.05). Comparisons among severity groups at T1 revealed that severe cases continued to have significantly higher TGF-β2 levels than mild (p = 0.004) and moderate cases (p = 0.02).

**Table 3:** Conjunctival transforming growth factor-β2 and vascular endothelial growth factor concentrations in study groups.

Cytokines	Controls (n = 10)	KCS (n = 33)	p-values (groups)
TGF-β2 (ng/mL)			
T0	82.52 ± 26.00	89.20 ± 12.60	0.07
T1	82.52 ± 26.00	108.50 ± 17.87	0.46
p-values (time points)	–	0.39	
VEGF (pg/mL)			
T0	20.30 ± 1.688	22.26 ± 1.30	0.44
T1	20.30 ± 1.688	18.92 ± 0.73	0.39
p-values (time points)	–	**0.002**	

Cytokine concentrations are expressed as mean ± SEM, statistical comparisons were performed using appropriate parametric or non-parametric tests depending on data distribution, p < 0.05 was considered statistically significant.Bold values are statistically significant (p < 0.05). Comparisons between groups (controls vs. KCS-affected eyes) were performed at both T0 and T1 using an unpaired t-test. Comparisons between time points (T0 vs. T1) within patients with KCS were analyzed using a paired t-test. KCS = keratoconjunctivitis sicca, TGF-β2 = transforming growth factor-β2, VEGF = vascular endothelial growth factor, SEM = standard error of the mean, T0 = baseline, T1 = post-treatment.

VEGF levels in the KCS population did not significantly differ from controls (p = 0.44) (Table 3, Figure 3b). However, VEGF levels declined significantly from T0 to T1 (p = 0.002). Severity-stratified evaluations showed no significant differences among mild, moderate, and severe groups at T0 (p = 0.76) or T1 (*p* = 0.12) (Table 4, Figure 4b). Paired comparisons revealed no significant differences for the mild and moderate groups, while a significant reduction in VEGF was observed in severe cases (p = 0.02) (Table 4, Figure 4b).

**Table 4:** Severity-based changes in conjunctival transforming growth factor-β2 and vascular endothelial growth factor levels before and after cyclosporine treatment in dogs with KCS.

Cytokines	Mild (n =10)	Moderate (n = 10)	Severe (n = 13)	p-values (KCS severity)
TGF-β2				
T0	30.14 ± 5.147	86.88 ± 16.69^a^	136.4 ± 22.11^b^	**0.004^a^, < 0.001^b^**
T1	48.09 ± 15.02^a^	79,27 ± 18.73^b^	177.5 ± 33.84	**0.004^a^, 0.02^d^**
p-values (time points)	0.18	0.33	0.11	
VEGF				
T0	23.19 ± 7.56	23.86 ± 9.53	20.30 ± 5.31	0.76
T1	19.27 ± 3.12	20.79 ± 6.01	17.25 ± 2.22	0.12
p-values (time points)	0.12	0.13	**0.02**	

Correlation coefficients were calculated using Pearson's or Spearman's correlation tests as appropriate; p < 0.05 indicates a statistically significant association. Bold values are statistically significant (p < 0.05). T0:

a Moderate and

b Severe significantly higher than mild. T1: ^a^Mild significantly lower than severe, ^b^Moderate significantly lower than severe. Comparisons among KCS severity groups (mild, moderate, and severe) were performed using one-way ANOVA (VEGF) with Bonferroni post hoc tests (TGF-β2). A paired t-test was used to assess within-group differences in changes in cytokine levels between T0 and T1. KCS = keratoconjunctivitis sicca, TGF-β2 = transforming growth factor-β2, VEGF = vascular endothelial growth factor.

Mean ± SEM concentrations of transforming growth factor-β2 (TGF-β2, ng/mL) (a) and vascular endothelial growth factor (VEGF, pg/mL) (b) in eyes with mild (n = 10), moderate (n = 10), and severe (n = 13) keratoconjunctivitis sicca (KCS) before treatment (T0) and after treatment (T1) with 0.2% cyclosporine.

At T0, cytokine levels in the moderate and severe KCS groups were significantly higher than those in the mild group. At T1, cytokine levels in the mild and moderate groups were significantly lower than those in the severe group, as indicated. Comparisons among KCS severity groups were performed using one-way analysis of variance with Bonferroni post hoc tests, and within-group changes between T0 and T1 were analyzed using paired t-tests. Bold values indicate statistically significant differences (p < 0.05). KCS = keratoconjunctivitis sicca, TGF-β2 = transforming growth factor-β2, VEGF = vascular endothelial growth factor, SEM = standard error of the mean, T0 = baseline, T1 = post-treatment.

### Relationship between TGF-β2, VEGF, STT-1, and ophthalmic parameters

**Figure 5:**
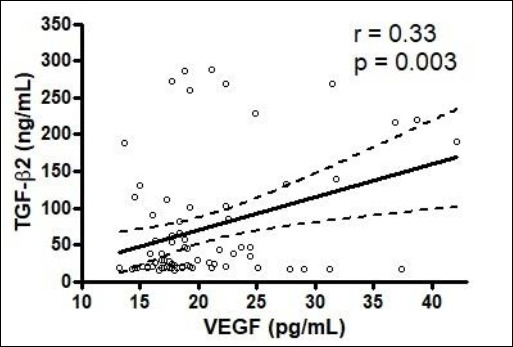
Relationship between conjunctival transforming growth factor-β2 and vascular endothelial growth factor levels in dogs with keratoconjunctivitis sicca. Linear regression analysis illustrating the relationship between conjunctival transforming growth factor-β2 (TGF-β2, ng/mL) and vascular endothelial growth factor (VEGF, pg/mL). The solid line represents the fitted regression line, and the dotted lines indicate the corresponding confidence intervals. TGF-β2 = transforming growth factor-β2, VEGF = vascular endothelial growth factor.

Table 5 summarizes correlations between cytokines and evaluated variables. TGF-β2 shows significant negative correlations with GCD (p = 0.003), neutrophils (p = 0.001), and total inflammatory cells (p = 0.02), along with a positive correlation with corneal melanosis (p = 0.02). VEGF is negatively correlated with corneal vessel branches (p = 0.02) and positively correlated with melanosis (p = 0.005). Additionally, conjunctival TGF-β2 correlates positively with VEGF (p = 0.003) (Figure 5).

**Table 5:** Correlations between transforming growth factor-β2 (TGF-β2 ng/mL) levels and vascular endothelial growth factor (VEGF pg/mL) levels with Schirmer tear test-1, ophthalmic clinical signs, goblet cell density, and inflammatory cells.

Parameters	TGF-β2 (ng/mL)	VEGF (pg/mL)
Schirmer tear test: 1	r = 0.07, p = 0.53	r = 0.10, p = 0.38
Ocular discharge	r = 0.03, p = 0.77	r = 0.13, p = 0.27
Conjunctival hyperemia	r = 0.03, p = 0.73	r = 0.08, p = 0.49
Corneal edema	r = 0.09, p = 0.23	r = 0.04, p = 0.70
Corneal vessel branches	r = 0.16, p = 0.15	**r = 0.50, p = 0.02**
Corneal melanosis	**r = 0.41, p = 0.02**	**r = 0.50, p = 0.005**
Goblet cell density	**r = -0.40, p = 0.003**	r = 0.08, p = 0.69
Lymphocytes	**r = 0.35, p = 0.04**	r = 0.08, p = 0.70
Plasma cells	r = 0.25, p = 0.15	r = 0.002, p = 0.99
Neutrophils	**r = -0.50, p = 0.001**	r = 0.07, p = 0.73
Total number of inflammatory cells	**r = -39, p = 0.02**	r = 0.07, p = 0.74

Pre- and post-treatment values are presented as mean ± SEM, statistical comparisons were performed using paired analyses, p < 0.05 was considered statistically significant. Bold values are statistically significant (p < 0.05). Spearman's test was used to assess correlations between TGF-β2 and VEGF levels and ophthalmic clinical signs, goblet cell density, and inflammatory cells. Pearson's test was used to evaluate the correlations between STT-1 and inflammatory cells. KCS = keratoconjunctivitis sicca, STT = Schirmer tear test, TBUT = tear film break-up time, CsA = cyclosporine A, SEM = standard error of the mean.

Linear regression analysis illustrating the relationship between conjunctival transforming growth factor-β2 (TGF-β2, ng/mL) and vascular endothelial growth factor (VEGF, pg/mL). The solid line represents the fitted regression line, and the dotted lines indicate the corresponding confidence intervals.

TGF-β2 = transforming growth factor-β2, VEGF = vascular endothelial growth factor.

## DISCUSSION

### Impact of 0.2% CsA on STT-1 expression, ophthalmic signs, inflammatory cell counts, and GCD

Despite the expected improvement in overall clinical signs in dogs with keratoconjunctivitis sicca (KCS) after treatment with topical immunosuppressants, this is the first study to demonstrate the effectiveness of 0.2% cyclosporine A (CsA) across all stages of canine KCS, with a possible influence on conjunctival transforming growth factor-β2 (TGF-β2) and VEGF levels. However, in all previous veterinary ophthalmology studies, the mild and severe forms of KCS were combined into a single severe category [2, 3]. Although CsA treatment tended to decrease conjunctival hyperemia, ocular discharge, corneal edema, and corneal vessel growth across all KCS severity levels, significant improvements in the first two signs were only seen in the mild form of the disease. Notably, corneal edema showed a significant reduction only in the moderate form, and corneal vascularization only in the severe form. Corneal melanosis and vascularization are often seen with conjunctival hyperemia [1–6]. This occurs because decreased tear production promotes dryness, which overrides the cornea’s innate anti-angiogenic defenses and damages its avascular nature [1–6].

Corneal melanosis in dogs with KCS is thought to occur due to melanocyte invasion of the corneal epithelium as the disease advances [1]. The results of this study supported this trend and showed that a 6-week treatment with 0.2% CsA did not significantly reduce melanosis at any stage of the disease. These results may partly explain the findings, as signs associated with chronicity, particularly corneal vascularization and melanosis, are likely to need longer periods to improve [3, 5].

GCD increased slightly after CsA treatment and reached levels similar to controls, although this was not statistically significant. While GCD tended to rise after 6 weeks of 0.2% CsA treatment, it did not reach statistical significance. However, the GCD values in CsA-treated eyes were comparable to those in control eyes. Izci *et al*. [4] reported a significant increase in GCD in dogs with KCS after 6 weeks of treatment with topical 2% CsA, possibly due to the higher drug concentration. Nevertheless, that study included only four dogs and lacked a control group [4].

It has been reported that plasma cells, lymphocytes, and neutrophils in conjunctival samples collected from Shih Tzus with idiopathic KCS increase as the disease progresses from mild to severe [8]. This is the second study to compare the infiltration of inflammatory cells in the bulbar conjunctiva of dogs with KCS after treatment with topical CsA [4]. As expected, all inflammatory cell types were significantly higher in the conjunctiva of dogs with KCS than in controls. Except for lymphocytes, numerical reductions were observed for other cell types, but only neutrophils were significantly reduced after 45 days of treatment with 0.2% CsA. This contrasts with previous findings in which plasma cells significantly increased after treatment with 2% CsA for 45 days [4]. In the same study [4], the number of ANAE+ T lymphocytes, CD4+ cells, and CD8+ cells decreased significantly following 2% CsA, while B lymphocytes did not. However, that study lacked a control group, did not report neutrophil counts, and showed inconsistencies between right and left conjunctival samples, likely due to type II statistical error [4].

In the present study, lymphocyte counts remained stable after 45 days of treatment with 0.2% CsA. Although CsA can improve clinical signs and Schirmer tear test-1 (STT-1) values within 45 days [3–5], it may not be enough to significantly affect lymphocyte levels, as studies with tacrolimus show it takes about 6 months to significantly change lymphocyte profiles [6]. In this study, neutrophils were the only inflammatory cells that decreased notably following CsA therapy. This decrease may be related to the higher bacterial load in the conjunctival sac of dogs with KCS compared to healthy dogs [22, 23], since the increase in STT-1 values caused by CsA likely helped restore lysozyme and immunoglobulins, which assist in controlling conjunctival microflora.

### Conjunctival VEGF levels, effects of 0.2% CsA therapy, and correlations with corneal vessels and melanosis

no previous studies have measured vegf levels in tears or on the ocular surface of dogs. in humans, one study showed higher vegf expression in inflamed corneas, specifically in epithelial cells, endothelial cells, limbal vascular endothelial cells, and some keratocytes, compared to healthy controls, indicating a possible role for vegf in corneal neovascularization [13]. however, none of the corneas in that study were from individuals with ded [13]. another study with 102 adult participants found significantly higher tear vegf levels in individuals with atopy; however, vegf was not linked to age, dry eye symptoms, corneal staining, tear film break-up time, stt-1, tear meniscus height, or conjunctival hyperemia [15]. these results differ from another study that reported higher vegf levels in platelet-rich plasma eye drops used in patients with ded associated with sjögren’s syndrome (ded-ss), which correlated with improved symptom scores and stt-1, suggesting vegf has a functional role in ocular surface health [17].

In this study, VEGF and TGF-β2 levels were measured in conjunctival tissue because collecting tears from dogs with severe KCS was not practical during the initial assessment. Although VEGF levels were higher in dogs with KCS compared to controls, the difference was not statistically significant. A notable decrease in VEGF levels was observed following treatment with 0.2% CsA. These results suggest that improvements in clinical signs were linked to a reduction in VEGF levels across all grades of KCS severity, although statistical significance was only achieved in severe cases.

An interesting observation in the present study was that the improvement in conjunctival hyperemia did not correlate with conjunctival VEGF levels, even though it was statistically significant in the mild form. In contrast, both VEGF levels and the number of corneal vessel branches decreased significantly only in severe KCS. This might be explained by the strong negative correlation between VEGF and corneal vessel branches. Similar findings have been observed in humans, where vascular endothelial cells of limbal vessels showed prominent VEGF expression [3], and where topical CsA was more effective than bevacizumab in reducing corneal vascularization in a rat chemical burn model [18].

In dogs with KCS, corneal neovascularization is believed to foster melanocyte migration into the cornea, potentially leading to blindness in severe cases. Notably, VEGF levels showed a positive correlation with corneal melanosis in this study, an association not previously identified. These findings imply that longer CsA treatment durations might be necessary to better evaluate VEGF-related changes in ocular signs. Supporting this, one study observed that eyes treated with 2% CsA had significantly lower clinical scores at week 12 compared to week 6 [5]. This aligns with the idea that chronic signs such as vascularization and melanosis need extended treatment periods to resolve [3, 5]. Although VEGF directly affects immune cell recruitment, activation, and cytokine production [16, 17], no relationship was found between VEGF and inflammatory cell types in this study.

### Conjunctival TGF-β2 Levels and Effects of 0.2% CsA Therapy

Findings from this study showed that conjunctival TGF-β2 levels in dogs with KCS were not significantly different from those in control dogs when looking at the overall population. However, more detailed analysis indicated that TGF-β2 levels tended to increase as KCS severity progressed. These findings suggest that, at least in idiopathic canine KCS, TGF-β2 may serve as a pro-inflammatory cytokine linked to tissue remodeling, fibrosis, and inflammation of the ocular surface.

This interpretation is supported by two *in vitro* studies [24, 25]. One study showed that TGF-β2 encourages the transdifferentiation of conjunctival fibroblasts into myofibroblasts, a crucial step in fibrotic remodeling, by increasing α-smooth muscle actin (α-SMA) and collagen type I expression [25]. Another study found that TGF-β2 influences inflammatory responses in human conjunctival epithelial cells, with higher levels of TGF-β2 and its receptor observed when these cells were exposed to TNF-α and IFN-γ. These results are consistent with reports of elevated TGF-β2 expression in the conjunctival epithelium of patients with moderate-to-severe DED [25].

Similarly, an *in vitro* study showed that TGF-β2 activates the Smad2/3 pathway in human pterygium fibroblasts, leading to myofibroblast transdifferentiation marked by increased levels of α-SMA, laminin-α2, laminin-γ1, and fibronectin, which are key markers of ocular surface fibrosis [26]. Notably, CsA suppressed these TGF-β2-induced pro-fibrotic markers *in vitro*. However, the 6-week CsA treatment used in this study did not change TGF-β2 levels in dogs with KCS. This may be because CsA, a calcineurin inhibitor that mainly suppresses interleukin-2 and reduces the recruitment of CD4+ and CD8+ lymphocytes [4, 27], does not directly target the cellular sources responsible for TGF-β2 expression.

Contrary to our findings, TGF-β2 expression is reported to be lower in humans with DED and increases after 6–12 weeks of therapy with topical 0.05% CsA [11]. However, differences in sampling and methodology make direct comparison difficult. In the study by Pflugfelder *et al*. [11], conjunctival samples were obtained through impression cytology and assessed using immunohistochemistry, whereas the present study measured TGF-β2 levels with ELISA from macerated conjunctival tissue. Another human study found no differences in tear TGF-β1 and TGF-β2 using a Luminex bead assay, yet increased TGF-β bioactivity was observed in DED, especially cases associated with SS [10]. These differences emphasize the need for species-specific and method-consistent assessments. Mouse studies also suggest that TGF-β2 primarily localizes to the basal conjunctival epithelium and stroma during wound healing [28], but its distribution in the conjunctiva and lacrimal gland of dogs with KCS remains unknown.

### Associations between conjunctival TGF-β2, GCD, inflammatory cells, corneal melanosis, and VEGF

In idiopathic canine KCS, GCD decreases as the disease progresses from mild to severe [7,8]. In this study, conjunctival TGF-β2 levels increased significantly from mild to severe stages, while GCD decreased accordingly. A significant negative correlation between these parameters suggests that elevated TGF-β2 may contribute to goblet cell loss. This supports mouse model findings showing that TGF-β2 inhibits goblet cell differentiation by repressing the transcription factor SAM-pointed domain containing ETS transcription factor, a key regulator of goblet cell development [29]. Conditional deletion of TGF-β receptor II in keratin-14-positive epithelia led to goblet cell expansion, whereas sustained TGF-β2 signaling suppressed goblet cell proliferation [29]. These results imply that chronic upregulation of TGF-β2 in KCS may hinder goblet cell differentiation via SPDEF repression, contributing to tear film instability. Further research with canine goblet cells is needed to confirm this mechanism.

In this study, all inflammatory cell types except plasma cells correlated with TGF-β2 levels, reinforcing the idea that inflammation may influence TGF-β2 expression in idiopathic KCS. The positive correlation between lymphocyte counts and TGF-β2 supports the concept that TGF-β2 may switch from an immunoregulatory to a pro-inflammatory role in chronic ocular surface disease [30]. A mouse model of experimental autoimmune KCS showed that disrupting TGF-β signaling in T cells reduced ocular inflammation and enhanced epithelial barrier function, likely by modulating Th17/Th1 immune responses [30].

Despite the lack of correlation between TGF-β2 and corneal vessel branches, this study identified a significant positive relationship between TGF-β2 and corneal melanosis. This finding suggests that TGF-β2 may play a role in additional chronic ocular surface changes beyond inflammation.

Another notable finding was the positive correlation between TGF-β2 and VEGF levels. This relationship is supported by evidence from other ocular tissues and species [31–33]. TGF-β2 stimulates fibroblast proliferation, granulation tissue formation, collagen deposition, and angiogenesis in conjunctival wound healing models [28]. In pterygium, TGF-β overexpression promotes neovascularization [25]. Experimental autoimmune uveoretinitis models show that both cytokines increase early in disease progression before structural tissue damage occurs [31]. *In vitro* studies demonstrate that TGF-β2 induces VEGF expression through multiple signaling pathways, including Smad-dependent and Smad-independent cascades (PI3K/AKT, MAPK), amplifying angiogenic signaling [32, 33]. This interplay may create a positive feedback loop that contributes to chronic inflammation, fibrosis, and pathological neovascularization in dogs with severe KCS.

### Limitations and future perspectives

One limitation of this study was the small number of control samples available for VEGF and TGF-β2 analyses, as well as for stratified comparisons. The limited number of histological slides also prevented stratified comparisons of GCD and inflammatory cells among severity groups. The study design included only a single evaluation time point in control dogs and lacked a placebo treatment group, which may have introduced bias, as a mixed-model analysis could not be performed. Although many correlations were weak to moderate, they offer meaningful insights into the behavior of VEGF and TGF-β2 in the eyes of dogs with KCS.

Although CsA lowered VEGF levels, future research should investigate additional therapeutic strategies to regulate TGF-β2 in canine KCS. These could include neutralizing antibodies, ligand traps, small-molecule inhibitors of TGF-β receptor/Smad signaling, and histone deacetylase inhibitors like trichostatin-A [34].

## CONCLUSION

This study offers new insights into the clinical and immunological responses of dogs with keratoconjunctivitis sicca (KCS) treated with 0.2% cyclosporine A (CsA) and is the first to independently assess mild, moderate, and severe forms of the disease. Treatment with 0.2% CsA for about 6 weeks significantly increased Schirmer tear test-1 (STT-1) values across all severity levels and improved conjunctival hyperemia and ocular discharge in the mild form. Although chronic eye changes, especially corneal vascularization and melanosis, showed limited response within the 6-week period, a notable reduction in corneal vessel branches was seen in severe KCS, indicating that extended or more advanced disease stages can still benefit from CsA-mediated control of ocular pathology.

Conjunctival GCD increased numerically after CsA therapy and reached levels similar to those in healthy controls, suggesting an early recovery of mucin-producing cell populations. Among inflammatory cells, neutrophils were the only cell type significantly reduced following treatment, indicating a partial yet meaningful immunomodulatory effect of 0.2% CsA. Importantly, conjunctival levels of VEGF decreased significantly after therapy, especially in severe cases, while transforming growth factor-β2 (TGF-β2) levels remained unchanged despite increasing across severity grades and showing a negative correlation with GCD and a positive correlation with corneal melanosis. These findings support the involvement of TGF-β2 in chronic ocular surface remodeling and identify VEGF as a measurable marker that responds to CsA therapy.

From a practical perspective, the results highlight the effectiveness of 0.2% CsA as an initial treatment for enhancing tear production and regulating inflammatory and angiogenic pathways in dogs with KCS. The cytokine patterns also suggest possible therapeutic windows for future treatments targeting TGF-β2 and VEGF in chronic and advanced stages of the disease. A key advantage of this study is the stratified assessment of KCS severity, enabling a clearer understanding of disease-specific responses to CsA, an approach not previously documented.

In conclusion, 0.2% CsA provides significant clinical and immunological benefits within a 6-week treatment period, especially in enhancing tear production and lowering VEGF levels in severe KCS. However, chronic corneal changes and TGF-β2-driven tissue remodeling might require longer treatment durations or additional targeted therapies. Further research with longer follow-up, larger control groups, and investigation of antifibrotic or anti-TGF-β strategies is necessary to improve long-term restoration of the ocular surface in canine KCS.

## DATA AVAILABILITY

Supplementary data can be made available from the corresponding author upon request.

## AUTHORS’ CONTRIBUTIONS

BER, APR, TBL, ARC, MPL, and LJAQ: Conducted the study. BER: Data curation, investigation, project admini-stration, and writing – review and editing. APR: Conceptualization, data curation, formal analysis, methodology, project administration, resources, supervision, and writing – review and editing. TBL: Data curation. ARC: Data curation. MPL: Performed the ELISA tests. LJAQ: Histology. All authors have read and approved the final version of the manuscript.
